# The Indispensable Roles of Microglia and Astrocytes during Brain Development

**DOI:** 10.3389/fnhum.2016.00566

**Published:** 2016-11-08

**Authors:** Kitty Reemst, Stephen C. Noctor, Paul J. Lucassen, Elly M. Hol

**Affiliations:** ^1^Swammerdam Institute for Life Sciences, University of AmsterdamAmsterdam, Netherlands; ^2^Department of Psychiatry and Behavioral Sciences, UC Davis MIND InstituteSacramento, CA, USA; ^3^Translational Neuroscience, Brain Center Rudolf Magnus, University Medical Center UtrechtUtrecht, Netherlands; ^4^Netherlands Institute for NeuroscienceAmsterdam, Netherlands

**Keywords:** microglia, astrocytes, brain development, glial cells, neurodevelopmental disorders

## Abstract

Glia are essential for brain functioning during development and in the adult brain. Here, we discuss the various roles of both microglia and astrocytes, and their interactions during brain development. Although both cells are fundamentally different in origin and function, they often affect the same developmental processes such as neuro-/gliogenesis, angiogenesis, axonal outgrowth, synaptogenesis and synaptic pruning. Due to their important instructive roles in these processes, dysfunction of microglia or astrocytes during brain development could contribute to neurodevelopmental disorders and potentially even late-onset neuropathology. A better understanding of the origin, differentiation process and developmental functions of microglia and astrocytes will help to fully appreciate their role both in the developing as well as in the adult brain, in health and disease.

## Introduction

For a long time, the study of brain development has largely focused on neuronal development. However, neurons develop closely together in time with neuroglia, suggesting important interactions and a functional role for glia cells in brain development. Glia are further highly conserved throughout evolution and are, except for the cerebellum, the most abundant cell type in almost all subregions of the mammalian brain, suggesting an important role for these cells (Pfrieger and Barres, 1995). Studies experimentally quantifying glial cell numbers in mammalian brains have shown that in general, at least 50% of all cells in the human brain are glia, with considerable differences between studies and between different brain areas and a ratio that may change with age (Pelvig et al., 2008; Azevedo et al., 2009; Herculano-Houzel, 2009; Lyck et al., 2009). For example, the glia to neuron ratio of the cerebral cortex is approximately 3.76 and for the cerebellum 0.23 (Azevedo et al., 2009). Although in many textbooks and articles it is mentioned that the human brain contains a significant higher percentage of glial cells than primate and rodent brains (Pfrieger and Barres, 1995; Kandel et al., 2000; Nishiyama et al., 2005), this view seems to be rectified now. Herculano-Houzel et al. (2006, 2013) show that in humans, primates and rodents, glia numbers are estimated to amount to 50% of all brain cells. Although the cellular composition of the brain seems to be conserved between rodents, primates and humans, the translational relevance of studies on rodent glia should always be treated with care.

Due to the clear definition of the basic principles and properties of a neuron, i.e., a cell with the ability to transmit electrical signals in the form of action potentials, it is quite easy to classify related cells that do not fulfill these criteria as glia. The first description dates from Virchow (1858), who described glia as passive neural elements and the “connective tissue” or “glue” of the brain. While this definition was commonly used for a long time, it is now well accepted that glial functions are far more complex than initially described. Glia are in fact actively involved in many aspects of the nervous system such as the formation, plasticity and maintenance of neural circuits, and are needed for neuronal survival and function (Allen and Barres, 2009).

**Figure 1:**
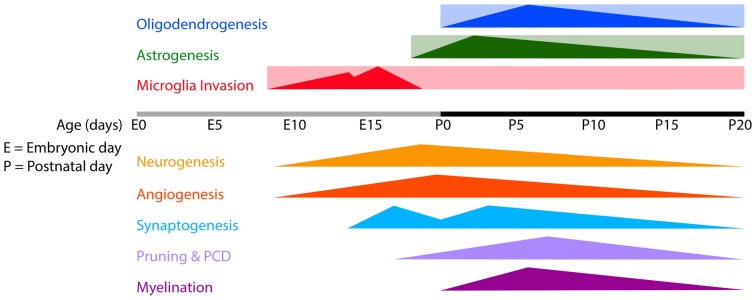
**Timeline of microglia invasion, gliogenesis and several developmental processes in the developing mouse brain.** Rectangles indicate the estimated periods during/from which microglia, astrocytes and oligodendrocytes remain present in the brain. Triangles indicate the onset and peaks of the indicated developmental processes. Abbreviations: E, embryonic; P, postnatal; PCD, programmed cell death.

Glia can be classified as different subsets, based on their morphology, function and location in the nervous system (Zhang, 2001). The two main glial subsets in the CNS are macroglia, including astrocytes and oligodendrocytes, and microglia. Macroglia are derived from the neural lineage and are produced after the initial neuronal birth wave. The origin of microglia used to be somewhat controversial, but now most evidence supports a “yolk-sac” macrophage-based source, from where they colonize the brain during prenatal development (Figure 1). While macroglia are commonly considered tissue-supporting cells, and microglia are considered the “immune cells of the brain”, more regulatory functions of these cells are emerging, as will be discussed below. In addition to macro- and microglia, neural stem cells can express glial markers and are often defined as glial subtypes that potentially perform distinct functions. In this review article, we focus on recent insights into the differential roles of microglia and astrocytes, particularly during early development of the brain.

Depending on the quantification method, microglia make up approximately 5–15% out of all cells in the human brain (Pelvig et al., 2008; Lyck et al., 2009). Initially, microglia were seen as “resting”, resident immune cells that only become activated, and start to phagocytose and secrete chemokines and cytokines, in response to pathological triggers. Generally, microglia were viewed as cells that help protect the brain against damage and infection (Gehrmann et al., 1995; Kreutzberg, 1996).

Recent evidence shows that microglia are highly dynamic cells. Under both physiological and pathological conditions, they scan their environment and regulate tissue homeostasis through scavenging functions (Davalos et al., 2005; Nimmerjahn et al., 2005; Raivich, 2005; Joly et al., 2009; Ransohoff and Brown, 2012). Apart from their well-known immune functions, microglia can influence synaptic transmission and synaptogenesis (Li et al., 2012; Pascual et al., 2012; Béchade et al., 2013) and can contribute to the maturation of neural circuits (Paolicelli et al., 2011; Cunningham et al., 2013; Kettenmann et al., 2013). Microglia are even thought to be able to sense and respond to local neural activity, due to their expression of most known neuro-transmitter receptors (Kettenmann et al., 2011) and their capacity to secrete neuroactive molecules (Lucin and Wyss-Coray, 2009).

In terms of terminology, it is important to keep in mind that the term “activated microglia” has been reconsidered. It is now believed that a change in activation state should be considered more a change in the functional phenotype, depending on the stimulus, than an activation or awakening (Hanisch and Kettenmann, 2007; Ransohoff and Perry, 2009).

Astrocytes are “star-shaped” cells that were first described in 1891 (Lenhossek, 1891), and represent the most abundant cell type in most parts of the brain (Nedergaard et al., 2003). In the 1990s they were recognized as active elements in the brain that can sense, integrate and respond to synaptic activity and can thereby contribute to brain homeostasis and neuronal function (Cornell-Bell et al., 1990; Porter and McCarthy, 1996; Kimelberg, 2004; Sofroniew and Vinters, 2010). Astrocytes share a common origin with neurons and oligodendrocytes, i.e., precursor cells derived from neuroepithelial cells, and are produced concurrently with the final stages of neurogenesis (Skoff, 1990; Figure 1). Once born, developing astrocytes differentiate into mature cells, as characterized by changes in morphology, connectivity and electrophysiological properties (Yang et al., 2013). Together with the pre- and postsynaptic parts of two neuronal synapses, astrocytes can form a so-called “tripartite” synapse, that helps maintain brain homeostasis and modulate synaptic transmission via the release of gliotransmitters such as glutamate, D-serine, and ATP (Dani et al., 1992; Araque et al., 1999; Newman, 2003; Kozlov et al., 2006; Wang et al., 2006; Halassa et al., 2007). Importantly, the increased cytosolic Ca2+ concentrations, e.g., as a response to neuronal activity, can activate other astrocytes and leads to a wave of calcium ions in the astrocytic network (Giaume et al., 1991).

Apart from communication via release of gliotransmitters, astrocytes also supply neurons with the substrates for neurotransmitters to enhance neural activity, communicate with neurons through neurotransmitter uptake and release and terminate the action of neurotransmitters by assisting in their recycling from the synaptic cleft (Pfrieger and Barres, 1997; Ullian et al., 2001).

## Microglia—The First Glial Cells to Enter the Brain

Microglia are the first glial cells observed in the brain, and develop side by side with neurons during the critical period of early embryonic brain development (Pont-Lezica et al., 2011). We therefore first discuss microglial cell origin, invasion and distribution throughout the embryonic mouse brain, unless otherwise stated.

### The Origin of Microglia

Despite intensive research on microglia, their origin has been a matter of debate (Cuadros and Navascués, 1998; Kaur et al., 2001; Streit, 2001; Rezaie and Male, 2002; Chan et al., 2007; Ginhoux et al., 2013; Tremblay et al., 2015). The cells were first described in the work of Nissl (1899), Robertson (1899) and Ramon and Cajal (1909) as “reactive glial elements”, “mesoglia” and “the third neural element”, respectively. And even though Robertson’s hypothesis ended up to be true, it was later proved that the “mesoglia” he described were actually oligodendrocytes (Gill and Binder, 2007). The name “Microglia” was coined by Del Rio-Hortega (1919, 1932), who described the cells as non-neuronal elements that were distinct from the neuroectodermal macroglia—the oligodendroglia and astroglia. Later work hypothesized that microglia originated from the subependyma adjacent to the lateral ventricles (Lewis, 1968), from blood vessel-associated pericytes (Mori and Leblond, 1969; Barón and Gallego, 1972) or from yolk-sac macrophages (Alliot et al., 1999). However, none of these options were sufficiently verified through experimentation and thus not generally accepted. It was also considered that microglia, like peripheral tissue-resident mononuclear phagocytes, were derived from bone marrow (BM) derived circulating blood monocytes. When it was noted that the first microglia enter the brain mainly prenatally, i.e., before the establishment of a robust brain vasculature, it was proposed that they derive from a specific subset of mesodermal progenitors independent of the monocyte lineage but the exact progenitor was not yet defined (Chan et al., 2007).

This issue appears to have been solved now because evidence is accumulating that opposes the monocytic theory and rather supports the yolk-sac theory. This theory, first proposed in 1999 (Alliot et al., 1999) and recently reviewed and experimentally confirmed (Ginhoux et al., 2010; Schulz et al., 2012; Kierdorf et al., 2013), proposes that microglia are derived from yolk-sac primitive myeloid progenitor cells. Yolk-sac derived macrophages invade the brain at early embryonic stages and eventually account for the vast majority of microglia in the adult. This has now been demonstrated in zebrafish (Herbomel et al., 2001), birds (Cuadros et al., 1993), rodents (Ashwell et al., 1989; Sorokin et al., 1992; Santos et al., 2008; Rigato et al., 2011; Swinnen et al., 2013) and humans (Rezaie et al., 1999; Rezaie, 2003; Monier et al., 2007; Verney et al., 2010). Whether at later time points, microglia of different origin also invade the brain parenchyma, remains subject of debate.

### Microglia Invasion into the Embryonic Brain

Migration into, and colonization of, the whole mouse embryo by yolk-sac derived macrophage precursors starts between E8 and E10 (Ginhoux et al., 2010; Schulz et al., 2012) and the brain is the first organ to be colonized (Sorokin et al., 1992; Cuadros et al., 1993; Herbomel et al., 2001). In mice, the first microglia progenitors can be detected in the brain around embryonic age E9 (Alliot et al., 1999; Ginhoux et al., 2010; Schulz et al., 2012), and at similar developmental stages in other species (Sorokin et al., 1992; Cuadros et al., 1993; Herbomel et al., 2001). Interestingly, in rodents, microglia invade the brain and spinal cord parenchyma simultaneously which coincides with different developmental stages in the two areas. Namely, a period during which neurogenesis comes to an end in the spinal cord, whereas it is only the onset of neurogenesis in the brain (Caviness et al., 1995; Götz and Barde, 2005; Rigato et al., 2011; Swinnen et al., 2013). Therefore, embryonic microglia may be able to exert different functions in the brain vs. the spinal cord, e.g., microglia may influence neuro-, glio- and angiogenesis in the brain, and could contribute to the development of the first functional neuronal networks in the spinal cord (Rigato et al., 2011). Here, we focus on invasion of microglia into the brain, but will also discuss some interesting findings on the distribution of microglia in the spinal cord.

Microglial invasion into the CNS is believed to occur in two waves (Chan et al., 2007; Rigato et al., 2011; Swinnen et al., 2013; Figure 1). In mice, the first wave occurs between E8.5 and E14.5 during which microglia progenitors start to colonize the brain and microglia numbers increase gradually (Alliot et al., 1999; Rigato et al., 2011; Swinnen et al., 2013). This first gradual increase in microglia is likely caused by a rapid proliferation of pre-entered microglia together with the invasion of some new microglia precursors (Monier et al., 2007; Swinnen et al., 2013). Around E9.5, the first capillary sprouts begin to invade the neuroepithelium (Mancuso et al., 2008; Vasudevan and Bhide, 2008; Vasudevan et al., 2008). As at this stage there is no vascular network yet, it has been proposed that during the first wave of microglia invasion, microglia invade the brain via extravascular routes (Kurz and Christ, 1998; Streit, 2001; Chan et al., 2007; Arnold and Betsholtz, 2013). There seem to be two routes by which embryonic microglia can enter the brain, but this is not well established yet. They enter either from the meninges by crossing the pial surface (PS) or from the ventricles, where they can be found as free-floating cells or attached to the ventricle wall, from where they are thought to cross the ventricle wall into the brain parenchyma (Sorokin et al., 1992; Navascués et al., 2000; Swinnen et al., 2013). In zebrafish it has been demonstrated nicely that microglia follow cues from apoptotic cells when they migrate into and distribute themselves in the embryonic brain (Casano et al., 2016; Xu et al., 2016). In rodent embryonic brains, microglia may also be found in areas of cell death (Ashwell, 1991; Swinnen et al., 2013), however whether apoptotic signals trigger microglia to migrate into the brain parenchyma has not been addressed yet in mammals.

Between E14 and E16, there is a second massive increase in microglia number. This rapid increase of brain microglia cannot be explained by proliferation alone, since the number of proliferating microglia actually decreased from E14.5 on Swinnen et al. (2013). Therefore, a new wave of microglia progenitors, which enter the brain from the periphery, is thought to contribute (Chan et al., 2007; Arnold and Betsholtz, 2013; Swinnen et al., 2013). Hereafter the number of microglia continue to increase, but more slowly, until E17.5, during which period they scatter throughout the brain (Santos et al., 2008; Swinnen et al., 2013).

Under physiological conditions, microglia proliferate throughout the period of embryogenesis and self-renew constantly throughout life to maintain their cell numbers, without a contribution from bone-marrow derived macrophages (Ajami et al., 2007; Hashimoto et al., 2013; Elmore et al., 2014). After specific, conditional ablation of microglia in adult mice, via a tamoxifen (TAM)-inducible Cre-recombinase expressed under the control of the C-X3-C motif chemokine receptor 1 (Cx3cr1) promotor (Cx3cr1CreER mice), the cells renewed themselves locally within a week through massive proliferation, mediated by interleukin-1 receptor (IL-1R) signaling (Bruttger et al., 2015). Interestingly, Elmore et al. (2014) identified a microglial progenitor cell in the adult mouse brain that is responsible for repopulation of the brain after depletion of all microglia using colony-stimulating factor 1 receptor (CSF1R) inhibitors. It seems that the newly formed microglia are true ramified microglia but a more specific characterization of these cells is needed. Interestingly, the observed progenitor cell was nestin positive, which raises the question how it is possible that microglia, of myeloid lineage, could arise from nestin positive progenitor cells, perhaps of neuroectodermal origin. In favor of this possibility, it has been proven possible to generate microglia *in vitro* from embryonic stem cells (ESCs). This is only possible when the ESCs first differentiate into a neuronal nestin positive stage after which neuronal growth factors are removed and the cells differentiate into microglia (Beutner et al., 2010). Also, microglia have been shown to be capable of expressing nestin in culture and after brain injury (Sahin Kaya et al., 1999; Yokoyama et al., 2004; Wohl et al., 2011). Alternatively, both myeloid and neuroectodermal lineage derived cells may share expression of the nestin intermediate filament.

Thus, embryonic microglia are thought to colonize the brain and retina before, and independent of, the establishment of a vascular system (Santos et al., 2008; Ginhoux et al., 2010; Rymo et al., 2011; Arnold and Betsholtz, 2013). Nevertheless, it is possible that during later stages of brain development, microglia enter the brain parenchyma through blood vessels. This notion is supported by experiments on Ncx1−/− mice, that lack a heartbeat and a functional blood circulation and have no microglia in the brain on a time point during which Ncx1+/+ mice do, suggesting that microglia travel through blood vessels into the brain (Koushik et al., 2001; Ginhoux et al., 2010). Others, however claim that these data do not demonstrate that microglia entering the brain through blood and microglia may use, or need, pial penetrating vessels to migrate along into the brain parenchyma (Arnold and Betsholtz, 2013). Nevertheless, several studies have demonstrated that bone-marrow derived circulating macrophages can enter the brain through blood vessels at least under inflammatory conditions (Simard and Rivest, 2006; Jung and Schwartz, 2012). However, it seems that infiltrating cells do not settle in the brain or integrate in the microglial network and are most likely of no contribution to the microglial pool (Ajami et al., 2011; Ransohoff, 2011).

### The Development and Distribution of Embryonic Microglia

**Figure 2:**
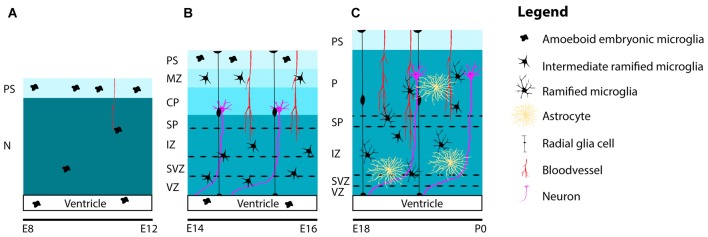
**Schematic representation of the distribution and maturation of microglia and the beginning of astrogenesis in the mouse cerebral cortex. (A)** At early embryonic stages between E8 and E12, microglia are located at the PS in the meninges and in the lateral ventricles. Only a few highly, motile and proliferative cells can be found in the neuroepithelium. **(B)** Between E14 and E16, microglia ramify further to form intermediate ramified microglia that can be found in the VZ, SVZ, IZ, SP and MZ. At the PS and in the lateral ventricle, one can still detect amoeboid microglia. Remarkably, until E16, microglia are absent from the cortical plate. **(C)** From E18 and onwards, microglia can be found in the cortical layers, after migrating from the deeper to the more superficial layers. During this period, microglia are found in close association with radial glia, blood vessels and developing axons. Around E18 astrogenesis starts. Similar to microglia, astrocytes are also found near radial glia, blood vessels and axons. Along the course of embryonic brain development, many microglia ramify further and transform from intermediate ramified microglia into mature ramified microglia with long processes. Abbreviations: PS, pial surface; N, neuroepithelium; MZ, marginal zone; CP, cortical plate; SP, subplate; IZ, intermediate zone; SVZ, subventricular zone; VZ, ventricular zone; P, parenchyma.

Together with microglia invasion, the patterns of colonization and distribution of microglia in the embryonic mouse brain have been studied well (Perry et al., 1985; Ashwell, 1991; Sorokin et al., 1992; Swinnen et al., 2013). Between E10 and E12, when the embryonic mouse-brain consists of mostly neuroepithelium, the first amoeboid microglia progenitors are observed at the PS in the meninges and within the lateral ventricles, where they can be found throughout the period of embryonic brain development. At these early embryonic stages, only a few proliferative and highly motile microglia can be detected in the neuroepithelium (Sorokin et al., 1992; Navascués et al., 2000; Swinnen et al., 2013; Figure 2A).

During embryonic brain development, amoeboid microglia transform into ramified microglia and the proportion of microglia with long processes increases with time (Swinnen et al., 2013; Figure 2). It is thought that the dynamic and mobile characteristics of microglia represent their ability to efficiently explore their environment (Herbomel et al., 2001; Nimmerjahn et al., 2005; Raivich, 2005; Swinnen et al., 2013). Swinnen et al. (2013) suggested that the observed increase in length of microglia processes over time reflects their current shape, and not only their maturation or activation state but may also indicate functional changes, e.g., to subsequent stressors or inflammatory challenges (Madore et al., 2013; Delpech et al., 2015). This is important to keep in mind when classifying microglia according to their phenotype.

Recently, three stages in microglia development have been identified (Matcovitch-Natan et al., 2016). The stages are classified as early (E10.5−E14), pre- (E14−P9) and adult (4 weeks and onwards) microglia, during which the cells express different sets of genes that reflect their stage related activities in the brain. Genetic and environmental perturbations caused changes in stage-related expression profiles and functions of microglia. The authors hypothesize that disturbances in the microglial environment can alter the precise timing of the microglial developmental programs, thereby disrupting brain development, and possibly causing neuropathology. In favor of this hypothesis, it was shown that the timing of prenatal infections, greatly affects its outcome on brain development and is thought to determine the specificity of behavioral pathology (Meyer et al., 2006a,b). Apart from temporal differences in gene expression profiles, region-specific differences have also been found (Doorn et al., 2015; Matcovitch-Natan et al., 2016). Also, distinct types of microglia were found to be present in different brain regions and have been implicated in neuropathological disorders such as Parkinson’s disease (Doorn et al., 2012, 2014, 2015), which could have a developmental origin. The above described studies suggest that together with the intrinsic properties of the microglia, local environmental factors determine the phenotypic characteristics and functions of microglia during embryonic development of the brain.

Neurons and microglia communicate with each other via ligand-receptor partnerships (Eyo and Wu, 2013). An example of such a partnership, that plays an important role in microglial development and activation state, is the inhibitory immune complex between neurons and microglia via the membrane glycoprotein Cluster of Differentiation 200 (CD200; also known as Ox2), constitutively expressed by neurons, and Cluster of Differentiation 200 receptor (CD200R), expressed by microglia (Wang et al., 2007). CD200/CD200R signaling is developmentally regulated and has been shown to keep microglia in a quiescent state, that is, not immunologically activated (Hoek et al., 2000; Lyons et al., 2007; Shrivastava et al., 2012). Another example is the expression of the chemokine C-X3-C motif ligand 1 (CX3CL1; also known as fractalkine) by neurons and its receptor CX3CR1 by microglia. In adult mice, CX3CL1/CX3CR1 signaling is involved in the regulation of microglial activation. Moreover, microglial cell-autonomous neurotoxicity was increased in CX3CR1 deficient mice (Cardona et al., 2006). It is likely that also in the developing brain, CX3CL1/CX3CR1 signaling is an important mechanism in preventing microglial toxicity, but this requires further investigation.

In order to study microglial function, phenotype and distribution, microglial markers have been identified. Examples are Ionized calcium Binding Adapter molecule 1 (Iba1) and the glycoproteins Cluster of Differentiation 11b, 68 and 45 (CD11b/68/45), that are used to visualize microglia and detect the cells in different functional activation states (see Korzhevskii and Kirik, 2016). Usually it is difficult to distinguish between CNS resident microglia and infiltrating monocytes since many microglial markers—proteins are expressed by blood-borne monocytes. Often, CD45 is used to distinguish microglia from monocytes, since under physiological conditions it is more highly expressed in monocytes as compared to microglia (Jeong et al., 2013; Ritzel et al., 2015). Also, the use of genetic mouse strains with green fluorescent protein (GFP) coupled to microglial specific promotors, such as CX3CR1, or BM chimeras, in which BM-derived macrophages are labeled with GFP, can be used to make clear distinctions between resident microglia and blood-borne monocytes (Jung et al., 2000; Jung and Schwartz, 2012).

During the course of embryonic brain development, microglia disperse from the neuroepithelium and are (re-) distributed in a non-uniform manner throughout the brain parenchyma in a dorsal-to-ventral and rostral-to-caudal gradient (Ashwell, 1991; Sorokin et al., 1992). It has been posited that CX3CL1/CX3CR1 signaling is somehow involved in the regulation of microglia infiltration, distribution and/or proliferation in the developing brain, since knockdown of the CX3CR1 in mice resulted in transient reduced numbers of microglia in the early postnatal hippocampus and delayed microglial influx in the somatosensory cortex (Paolicelli et al., 2011; Hoshiko et al., 2012). Also, neural progenitor cells are thought to play an important role in the migration and positioning of microglia in the prenatally developing cortex via the secretion of C-X-C motif chemokine 12 (Cxcl12; Arnò et al., 2014). The authors demonstrated that the migration of microglia, expressing the Cxcl12 receptor C-X-C motif chemokine 4 or 7 (CxcR4 or CxcR7), into the ventricular zone (VZ) and subventricular zone (SVZ) was controlled by Cxcl12- expressing intermediate (basal) progenitors. Other, yet to be discovered mechanisms are likely to play a part in the guidance of microglial distribution over the embryonic brain. Interestingly, until approximately E16.5 microglia do not enter the cortical plate (CP; Sorokin et al., 1992; Swinnen et al., 2013; Squarzoni et al., 2014). Before colonization of the CP, microglia are detected in the ventricular and intermediate zones (IZ; Swinnen et al., 2013), regions containing the neural progenitor cells (Figure 2B). Around E17, microglia gradually begin to invade the CP starting with the deeper layers, during this process they also become more ramified (Figure 2C).

In contrast to the rather equal distribution of microglia over the adult brain, embryonic microglia are unevenly distributed and located in at least four specific “hotspots” in the brain (Perry et al., 1985; Ashwell, 1991; Cuadros et al., 1993; Verney et al., 2010; Pont-Lezica et al., 2011; Swinnen et al., 2013; Squarzoni et al., 2014). One hotspot is near the radial glia in the VZ and SVZ, where the cells have been described to play a role in the regulation of the size of the precursor cell pool (Cunningham et al., 2013). Second, microglia are often associated with newly forming blood vessels, where they could contribute to angiogenesis (Cuadros et al., 1993; Dalmau et al., 1997a; Monier et al., 2007). Third, phagocytizing microglia are found near dying cells in the choroid plexus and in the developing hippocampus, carrying out phagocytic activities (Ashwell, 1991; Dalmau et al., 1998; Swinnen et al., 2013). And fourth, microglia are found near developing axons (Cuadros et al., 1993; Ueno et al., 2013; Squarzoni et al., 2014), and are possibly involved in developmental axonal pruning and/or axonal growth and guidance mechanisms.

It is important to note that sex differences in microglial cell numbers, morphology and distribution have been reported in the developing rat brain (Schwarz et al., 2012; Lenz et al., 2013). Therefore, it should be kept in mind that sex-dependent mechanisms could influence microglial function during brain development. This could possibly contribute to the sex-dependent susceptibilities to certain developmental psychiatric disorders (Bao and Swaab, 2010; Lenz and McCarthy, 2015).

We have covered some studies describing the invasion and distribution of embryonic microglia. However, further investigation is needed to elucidate the precise signals mediating the direction, speed and eventual distribution of microglia over the embryonic brain. The distribution of embryonic microglia gives a hint of their functions in the developing brain, and will be discussed throughout this review. First we will discuss the origin, development and distribution of astrocytes in the developing brain.

## Astrocytes—The Second Glial Cells Present in the Brain

Astrocytes are the most abundant cell type in the brain and have many important physiological functions. They are largely produced during the final stages of neurogenesis. Some excellent reviews have been published about the switch from neurogenesis to astrogenesis and astrocyte development in general (Freeman, 2010; Chaboub and Deneen, 2012; Kanski et al., 2014; Molofsky and Deneen, 2015), but our understanding of their generation, development and maturation is still far from complete.

### The Origin(s) and Development of Astrocytes

Astrocytes originate from the neural lineage, and a wave of astrogenesis starts toward the end of the neurogenic wave (Skoff, 1990; Noctor et al., 2004, 2008). In mice, astrogenesis starts around embryonic age 18 (E18) and lasts at least until approximately postnatal day 7 (P7; Figures 1, 2C). However, similar to adult neurogenesis, cases of adult astrogenesis have been reported (Zhao et al., 2007). Initially, a homogenous pool of naïve neural precursors (NPCs) in the embryonic tube, also called neuroepithelial precursor cells (NEPs), transform into radial glia, i.e., pluripotent neural stem cells located in the VZ, that sequentially generate neurons and macroglia (Malatesta et al., 2000, 2003; Sauvageot and Stiles, 2002; Kriegstein and Noctor, 2004; Miller and Gauthier, 2007). When neurogenesis comes to an end, radial glia can either differentiate directly into astrocytes, or produce intermediate cells that later become astrocytes.

Once born, developing astrocytes differentiate into mature ones, a process characterized by changes in morphology, connectivity and electrophysiological properties (Yang et al., 2013). Evidence is accumulating that astrocytes are a heterogeneous cell population with differences between cells across brain regions, as well as within the same brain regions (Hewett, 2009; Zhang and Barres, 2010). Since their discovery, astrocytes have been divided into two groups; protoplasmic and fibrous astrocytes, located in the gray and white matter, respectively (Kölliker, 1889; Andriezen, 1893). Over one century ago morphological differences within groups of astrocytes were noted (Ramon and Cajal, 1909). Spheroid shaped protoplasmic astrocytes have more complex branched extensions as compared to fibrous astrocytes which have long extensions that are longitudinal oriented along fiber bundles (Molofsky et al., 2012). Today we know that the rough classification of protoplasmic and fibrous astrocytes is likely to underestimate the number of distinct subtypes of astrocytes, that can differ in morphology, gene expression profile and physiological properties. These different subsets imply functional diversity of astrocytes (Zhang and Barres, 2010).

Just like different neuronal subtypes originate from distinct progenitors, different subtypes of astrocytes are thought to arise from distinct progenitors (Zhang and Barres, 2010). In the spinal cord, several studies have demonstrated functional subtypes of astrocytes in spatially different domains (Muroyama et al., 2005; Hochstim et al., 2008; Tsai et al., 2012). Moreover, Tsai et al. (2012) have demonstrated that spinal cord astrocytes have specific embryonic sites of origin in the VZ. Functional loss after depletion of region-specific astrocytes could not be rescued by migration of astrocyte precursors from other regions.

Our understanding of the precise steps in the maturation from progenitor cells into (a distinct group of) astrocytes is incomplete (Molofsky and Deneen, 2015). Some of the difficulties involved, are: (1) the lack of reliable and specific markers to define progenitor cells and immature astrocytes during their developmental stages; and (2) the difficulty to specifically manipulate genes that only affect astrogenesis and not neurogenesis, due to the fact that the so far identified astrocyte promoters are also active in neural stem cells (Chaboub and Deneen, 2012; Molofsky and Deneen, 2015). In addition, Chaboub and Deneen (2012) mention a third limiting factor for studying astrocyte development; i.e., the idea that the cells do not have a precisely defined developmental endpoint because adult astrocytes are mitotic cells and can in principle continue to divide and differentiate. However, it is also possible to look at this differently; in general there is a clear endpoint in astrocyte differentiation but the cells can use their mitotic potential and differentiate when needed, e.g., after injury, or in specialized areas such as the SVZ and sub granular zone (SGZ) where the cells serve as stem cells (Sofroniew, 2009). Additional research needs to be done in order to find out by which molecular mechanisms distinct subtypes of astrocytes are specified, whether these derive from distinct groups of progenitor cells, and how they develop to their diverse and complex morphologies.

### The Distribution of Astrocytes in the Neonatal Brain

While there is some data on the distribution of astrocytes in the adult mouse or rat brain, studies specifically describing their neonatal distribution and migration patterns are lacking (Bignami and Dahl, 1973; Kálmán and Hajós, 1989). This could be due to the lack of a general astrocyte marker. Taft et al. (2005) analyzed the distribution of astrocytes positive for one of the two main intermediate filaments expressed by astrocytes; glial fibrillary acidic protein (GFAP), used as a general astrocyte marker (Eng et al., 2000), and Vimentin, used as an embryonic astrocyte marker (Dahl et al., 1981). They found that in the neonatal rat brain GFAP+ and vimentin+ cells were distributed similarly and cells were located in high numbers throughout the whole brain, except the brainstem. This early postnatal distribution was similar to the adult distribution, suggesting that astrocytes distribute themselves right after their birth and do not change their location under physiological conditions throughout adulthood.

It has to be taken into consideration that although GFAP is a well-documented astrocyte marker, it is not always expressed at high levels by all astrocyte subtypes especially by astrocytes found in the gray matter (Walz and Lang, 1998; Walz, 2000). Also, other cell types such as radial glial cells and ependymal cells can express GFAP (Levitt and Rakic, 1980; Molofsky and Deneen, 2015). The same is true for Vimentin, a ubiquitous intermediate filament expressed by most mesenchymal cells and blood vessels (Satelli and Li, 2011). When studying astroglial development in the ferret, it was further found that there is a transition from vimentin to GFAP expression over a period of weeks (Voigt, 1989). Later studies in rat showed that as radial glial cells translocate out of the VZ toward the overlying CP, they begin to express GFAP (Noctor et al., 2004), further supporting the connection between radial glial cells and some astroglial cells. Moreover, in the adult brain, neural stem cells in the SVZ and SGZ also express GFAP (Alvarez-Buylla et al., 2002), indicating that they are a subclass of astrocytes or remnant radial glia. Besides classification by GFAP or Vimentin expression and the spatial or temporal appearance of astrocytes in the brain it could be useful to use other specific markers in order to identify subpopulations of astrocytes. Examples of other proteins expressed by astrocytes are calcium binding protein β (S100β), glutamine synthetase (GS), aldehyde dehydrogenase one family, member L1 (Aldh1L1), glutamate aspartate transporter (GLAST) and GFAP isoforms (Bachoo et al., 2004; Cahoy et al., 2008; Middeldorp and Hol, 2011; Mamber et al., 2012; Orre et al., 2014). The presence of these proteins is likely to underlie subtype-specific functions and knowledge about expression patterns will help understanding the different stages of astrogenesis and the distinct astrocytic phenotypes that are being observed.

Apart from using immunohistochemical methods, mice expressing GFP under the human GFAP or GLAST promotor (hGFAP-GFP or GLAST-GFP mice), have been proven to be a very useful tool to study the role of astrocytes and their lineage in the brain (Bardehle et al., 2013; Kim et al., 2013; Kraft et al., 2013; Ponti et al., 2013).

As discussed above, astrocytes are distributed over all areas of the CNS and they most likely migrate to their final destination shortly after their birth in the VZ or SVZ, considering of the observed similarities between the embryonic and adult distribution. Cortical gray matter astrocytes were found to migrate along radial glia processes, whereas white matter astrocytes migrated along developing axons of neurons (Bignami and Dahl, 1973; Jacobsen and Miller, 2003). As radial glial cells lose their processes during early postnatal stages, the question was raised as to how later VZ- or SVZ-born astrocytes can migrate into the cortical layers (Weissman, 2003). However, most postnatal born astrocytes seem to be generated through local proliferation of differentiated astrocytes (Ge et al., 2012).

Astrocytes can also be found located in close proximity of developing blood vessels and wrapped around developing synapses (Stone et al., 1995; Araque et al., 1999; Abbott, 2002; Dorrell et al., 2002). Thus similar to microglia, developing astrocytes can be found throughout the developing brain and in proximity to radial glia, blood vessels and axons (Figure 2C). The next sections will focus on the functions related to the distribution of both cell types in the developing brain.

## The Crosstalk Between Microglia, Neural Progenitors and Astrocytes

**Figure 3:**
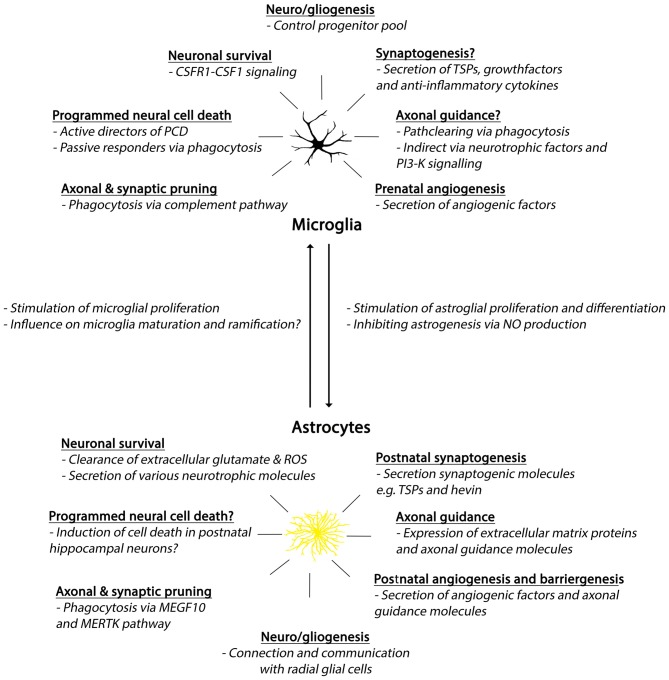
**Summary of developmental roles of microglia and astrocytes.** Abbreviations: CSFR1, colony-stimulating factor 1 receptor; CSF1, colony-stimulating factor 1; PCD, programmed cell death; TSPs, thrombospondins; PI3-K, phosphoinositide-3 kinase; ROS, reactive oxygen species; MEGF10, Multiple EGF-like-domains 10; MERTK, MER Tyrosine Kinase.

Microglia, astrocytes and neural progenitors can influence each other’s development and behavior in several manners. Here, we will give a few examples of such interactions (Figure 3). As described above, embryonic microglia migrate towards zones of active proliferation, possibly attracted by Cxcl12-expressing neural progenitors (Arnò et al., 2014). The localization of microglia close to proliferative zones suggests they can influence the neural progenitor pool and it has indeed been demonstrated *in vitro*, that cultured neural precursors when depleted of microglia, show decreased rates of proliferation and astrogenesis (Walton et al., 2006; Antony et al., 2011). Also in the developing cerebral cortex of macaques and rats, microglia can phagocytose intermediate precursors in the SVZ, thereby affecting neuro- and gliogenesis (Cunningham et al., 2013). A subset of microglia was further shown to produce nitric oxide (NO), a signaling molecule that regulates the developmental switch from neuro- to astrogenesis and astroglial maturation (Peunova and Enikolopov, 1995; Béchade et al., 2011). Interestingly, apart from their effect on developmental neurogenesis, microglia can influence adult hippocampal neurogenesis by phagocytosis of apoptotic cells (Sierra et al., 2010) and are also able to provide trophic support to new adult born neurons in the hippocampus and SVZ (for review see Gemma and Bachstetter, 2013; Ribeiro Xavier et al., 2015). It is thus likely that the mechanisms microglia use to affect developmental neuro- and astrogenesis, can also be used to influence SVZ and hippocampal neurogenesis in the adult (Aarum et al., 2003; Kanski et al., 2014; Valero et al., 2016).

If microglia can affect the neural progenitor pool, they could possibly play a role in astrogenesis as well. Once born in the VZ or SVZ, astrocytes are distributed over different brain regions where they differentiate into mature astrocytes. This, notably, only occurs after the appearance of microglia in that area. A delay is present between the appearance of microglia and astrogenesis and astroglial maturation in the human subplate and IZ, as well as in the mouse IZ and hippocampus (Matthias et al., 2003; Zhou et al., 2006; Béchade et al., 2011). The subsequent appearance of these two cell types closely after each other has led to the idea that microglia might regulate astrocyte differentiation, or the switch from neuro- to astrogenesis (Dalmau et al., 1997a; Rezaie, 2003). In support of such a concept, microglia depletion in cortical precursor cell cultures was shown to result in decreased numbers of newly generated astrocytes (Antony et al., 2011). Also, microglia are known to secrete several factors that can stimulate astrocyte proliferation and/or differentiation (Giulian et al., 1988; Nakanishi et al., 2007; Béchade et al., 2011).

On the other hand, microglial full maturation and ramification takes place rather late during development, when astrocytes are already present (Rezaie et al., 2002; Figure 2C). Therefore, it has been hypothesized that differentiated astrocytes might contribute to microglia maturation (Tanaka and Maeda, 1996; Navascués et al., 2000; Rezaie et al., 2002) and indeed, astrocytes can secrete soluble factors that can stimulate proliferation of microglia (Frei et al., 1986; Lee et al., 1994) but more experimental evidence for this hypothesis is lacking.

## Glial Influence on Developmental Angiogenesis

The formation of new blood vessels occurs via two distinct mechanisms, vasculogenesis and angiogenesis. During vasculogenesis vessels are formed *de novo* from differentiating and migrating angioblasts. The brain vasculature develops exclusively via angiogenesis, during which new blood vessels are formed from pre-existing ones. In the brain this process starts around E9.5, when the first vascular sprouts invade into the neuroepithelium with a caudal to cranial direction which is followed by branching, arborization and migration of capillary sprouts from the PS toward the ventricles where angiogenic factors like vascular endothelial growth factor (VEGF) are highly expressed (Risau, 1997; Patan, 2000; Vasudevan et al., 2008).

During this period, microglia are found in close association with developing blood vessels, directly after they enter the neuroepithelium and throughout CNS development, suggesting a potential role for microglia in blood vessel formation (Herbomel et al., 2001; Monier et al., 2007; Pont-Lezica et al., 2011). Although astrocytes appear much later in the brain during development, they too have been described to play a part in postnatal developmental angiogenesis. Moreover, after the establishment of the primary brain vasculature, astrocytes contribute to the formation of the blood—brain barrier (BBB), also called barrier-genesis.

### The Potential Role of Microglia in Vascular Network Formation

During development of the brain vasculature the first embryonic microglial progenitors are present in the brain and more cells keep invading (Vasudevan and Bhide, 2008; Vasudevan et al., 2008; Arnold and Betsholtz, 2013; Figure 1). Due to localization of microglia close to developing vasculature it has been suggested they may contribute to its formation (Cuadros et al., 1992, 1993; Dalmau et al., 1997b; Rezaie et al., 1999; Monier et al., 2007; Arnold and Betsholtz, 2013). Indeed, microglia depletion in the developing CNS results in a sparser vascular network (Checchin et al., 2006; Fantin et al., 2010; Rymo et al., 2011). In aortic ring cultures, microglia quickly migrate towards developing blood vessels and stimulate sprout formation and branching (Rymo et al., 2011). Rymo et al. (2011) show nicely that the stimulating effect of microglia in aortic ring cultures on vessel sprouting is mediated by microglial-derived soluble factors, rather than direct contact with endothelial cells. It is not clear from this study which angiogenic soluble factor(s) is secreted by microglia. However, it seems to be distinct from VEGF-A, as the experimenters show that inhibition of VEGF-A did not change microglial-induced vessel branching. Future research is necessary to investigate the factors that lead to attraction of microglia to developing vessels and mediate the following angiogenic properties of microglia.

### Astrocytes Contribute to Postnatal Angiogenesis and BBB Formation

The generation of the first astrocytes, takes place after the formation of the first blood vessels that invade the parenchyma from the PS (Figure 1). Nevertheless, astrocytes appear in a phase during which vessel sprouting is still taking place. During retinal development, astrocytes were shown to be actively involved in the formation of the retinal vasculature (Stone et al., 1995), where they provide a template over which endothelial cells can migrate and form the vasculature (Dorrell et al., 2002). Moreover, it has been implicated that astrocytes promote proper cortical blood vessel development in the developing brain. Perinatal inhibition of astrogenesis resulted in a drastic reduction in the density and branching of cortical blood vessels (Ma et al., 2012). Astrocytes further express VEGF, which was proposed to be necessary for the formation and stabilization of developing blood vessels (Alon et al., 1995; Stone et al., 1995; Scott et al., 2010). Some claim however that in the retina astrocyte-derived VEGF is only important in hypoxia-induced pathological angiogenesis and not in developmental angiogenesis under physiological conditions (Weidemann et al., 2010). In addition to VEGF, astrocytes were shown to be capable of secreting other angiogenic molecules such as angiopoietin-1 (Ang-1), angiopoietin-2 (Ang-2), endothelin-1 (ET-1) and oxygenase-1 (HO-1; Nakamura-Ishizu et al., 2012; Li et al., 2013; Hammond et al., 2014; Chen-Roetling et al., 2015). Apart from the secretion of angiogenic molecules, astrocytes are capable of expressing axonal guidance molecules and growth factors, as described in chapter 5.2, of which some were shown to affect vessel sprouting as well (Basile et al., 2004; Lu et al., 2004; Torres-Vázquez et al., 2004; Carmeliet and Tessier-Lavigne, 2005). It remains to be investigated if and how the secretion of these molecules is involved in developmental angiogenesis.

After the primary vascular network is established, brain vessels undergo barrier-genesis, during which the BBB is formed (Lee et al., 2009). The BBB is a diffusion barrier that is formed by endothelial cells connected by tight junctions with selected permeability. The endothelial cells are lined up along the cerebral microvasculature that protects the brain from circulating agents and fluctuations in plasma composition that could disturb neural function (Abbott and Romero, 1996; Abbott, 2002). Astrocytic end-feet form “rosette”-like structures lying on the vessel walls, that allow free diffusion between the endothelial cells and the brain parenchyma (Kacem et al., 1998; Abbott, 2002).

Astrocytes are further thought to play a role in BBB formation (Abbott et al., 2006, 2010). Most studies on BBB development and function are performed using *in vitro* systems (Dehouck et al., 1990; Rubin et al., 1991), and strongly suggest that astrocytes can induce BBB formation via the secretion of astrocyte-derived soluble factors (Beck et al., 1984; Neuhaus et al., 1991; Ballabh et al., 2004). However, some aspects of the BBB, such as tight junctions, are present before astrocytes appear in the brain (Haseloff et al., 2005; Saunders et al., 2008). Future studies are needed to address which aspects of the BBB are dependent on astrocytes and which become functional before astrocytes appear in the brain.

## The Role of Glia in Axonal Outgrowth and Guidance

During CNS development, the outgrowth of axons is guided by local neuronal and glial derived soluble factors that mediate the attraction or repulsion of the growth cone, a specialized sensorimotor structure at the axonal tip (Goodman, 1996; Dickson, 2002). The growth cone navigates from one intermediate target, a neuron or glial cell sending out guidance cues, to the next until reaching and innervating the final target (Bentley and Caudy, 1983; Ghosh and Shatz, 1993; Bailey et al., 1999). Most work on axonal guidance has been done in invertebrates because of the accessibility of their nervous system (Ito et al., 1995). During early embryonic CNS development in the drosophila, glial cells were found to express the chemo-attractant netrin (Jacobs, 2000) and the chemorepellents Slit and semaphorin (Kidd et al., 1999), all three necessary for accurate axon navigation and midline crossing of commissural axons (Kidd et al., 1998). In vertebrates, glial-like floor plate cells of the spinal cord, Cajal-Retzius cells, subplate cells, and oligodendrocytes precursor cells (OPCs) have been suggested to take part in this prenatal guidance role (Cavalcante et al., 2002; Goldberg et al., 2004), but a role for microglia and astrocytes cannot be excluded either and will be discussed here.

### In Which Ways are Microglia Involved in Axonal Outgrowth?

In various brain regions in different species such as rodents (Ashwell et al., 1989; Pont-Lezica et al., 2014), birds (Cuadros et al., 1993), zebrafish (Herbomel et al., 2001), cats (Innocenti et al., 1983) and humans (Rezaie et al., 1999; Verney et al., 2010), microglia are found to be closely associated with developing axonal tracks. For example, in rodents microglia were found in developing marginal zones (MZ) that contain developing axon fascicles (Cuadros et al., 1993; Soria and Fairén, 2000), the subpallium (Squarzoni et al., 2014), the corpus callosum (Pont-Lezica et al., 2014) and the hippocampal commissure (Dalmau et al., 1997a, 1998). Microglia associated with white matter acquire a distinct morphology which differs from the microglia located in gray matter. They seem to line up parallel to axonal tracks and keep this alignment during their ramification and maturation process (Cuadros et al., 1993; Dalmau et al., 1998; Torres-Platas et al., 2014). It is so far not known how this aligned structure contributes to microglial functions in association with axons.

Microglia have been extensively studied for their role in axonal pruning, but rarely in the process of axonal survival, outgrowth, or navigation. In accordance with their phagocytic functions, the first studies pointed towards a role for microglia in “path-clearing” for developing axons (Valentino and Jones, 1982; David et al., 1990) or the elimination of transient axonal projections (Innocenti et al., 1983). Microglia activated by lipopolysaccharide (LPS) express repulsive guidance molecule a (RGMa) thereby inhibiting axonal outgrowth and inducing collapsing of the growth cone (Kitayama et al., 2011). It has not been investigated if microglia during development express RGMa. Microglia also associate with developing axons in the absence of axon degeneration (Cuadros et al., 1993), thus a more trophic role for microglia in neurite extension should not be excluded. It has been demonstrated that in mice, at E14.5, the period of mid-neurogenesis, microglia are concentrated at and associated with dopaminergic axonal tracts that entered the subpallium, while they did not associate with adjacent serotoninergic or internal capsule fibers (Squarzoni et al., 2014). In addition, the researchers studied the effect of microglia on axonal outgrowth. Three mouse models were used: (1) mice depleted from most microglia via the blockade of the CSF-1R; (2) mice lacking all myeloid cells via knockout of Pu.1; and (3) mice that underwent maternal immune activation using peritoneal injection of liposaccharide (LPS) resulting in immune activated microglia. Mouse brains without microglia as well as with immune activated microglia, displayed abnormal dopaminergic axon outgrowth, as was visible by exuberant or reduced axonal extensions respectively, while other axons remained unaffected. It is not clear whether these abnormal extensions are a consequence of a failure in axonal guidance that caused uncontrolled axonal outgrowth or changes in axonal pruning. The latter seems likely since microglia can phagocytose fragments of dopaminergic axons and *Cx3cr1* negative mice, known for their deficits in microglia-neuron signaling and synaptic pruning, displayed a similar phenotype (Squarzoni et al., 2014). However, the authors also show that complement receptor 3 (CR3) and DNAX activation protein of 12 kDa (DAP12), both linked to synaptic pruning (Paloneva BM et al., 2001; Schafer et al., 2012), were not involved in the affected developmental process.

The largest commissure of the mammalian brain, the corpus callosum, has also been investigated in regard to microglial effects on its development (Pont-Lezica et al., 2014). The authors show that disruption of microglial function or depletion of microglia resulted in the defasciculation of dorsal callosal axons, suggesting that microglia are somehow involved in shaping callosal axonal tracts. Three animal models, in which microglia mediated phagocytosis was increased, impaired or even absent, all resulted in defasciculation of callosal axons. This suggests that defasciculation is not exclusively dependent on the phagocytotic properties of microglia. Microglia might also participate in axon growth and guidance via trophic support.

During brain development, microglia secrete several neurotrophic factors such as nerve growth factor (NGF), brain-derived neurotrophic factor (BDNF), neurotrophin-3 (NT-3), fibroblast growth factor (FGF), and insulin-like growth factor 1 (IgF-I), all of which control the activity of receptor tyrosine kinase and associated signaling through phosphoinositide-3 kinase (PI3-K). PI3-K was initially known for its role in cell survival but it has become clear that it also plays a role in axon development, elongation, and maintenance (Pap and Cooper, 1998; Sanchez et al., 2001; Shi et al., 2003). Thus, microglia might not directly guide axonal growth and guidance, but rather indirectly via PI3-K signaling.

The above described studies give a little bit more insight into the possible trophic effects of microglia on axonal outgrowth. However, future research is needed to understand why microglia would associate with and affect certain axonal tracts more as compared to others and what the underlying mechanisms are via which microglia can affect axonal outgrowth and guidance.

### Astrocytes Can Affect Axonal Outgrowth

Similar to microglia, astrocytes are found in high numbers in the white matter (Miller and Raff, 1984). Astrocytes have been reported to form molecular boundaries by which they guide neurite extension (Powell and Geller, 1999). During brain development, astrocytes express laminin and fibronectin, two glycoproteins of the extracellular matrix that are involved in axon elongation and pathfinding via contact-mediated attraction of the growth cone (Liesi and Silver, 1988; Tonge et al., 2012). Neuronal extracellular lysophosphatidic acid (LPA), a membrane-derived lysophospholipid, can stimulate astrocytes to induce axonal outgrowth of cortical progenitors by upregulation of laminin and fibronectin expression, possibly by the activation of mitogen-activated protein kinase (MAPK) and protein kinase A (PKA) pathways (De Sampaio E Spohr et al., 2011; Spohr et al., 2014). The chemoattractant netrin-4 was found to be expressed by a subset of astrocytes (Staquicini et al., 2009). However, astrocytic netrin-4 was only shown to influence neural stem cell migration and proliferation and it is unclear it also stimulates axonal outgrowth during development. Also, after ischemia, astrocytic feet were found to express the netrin receptor deleted in colorectal cancer (DCC; Tsuchiya et al., 2007). It is so far unclear whether astrocytic expression of netrin or its receptor is involved in developmental axonal outgrowth.

Also, ephrins and their receptors (Ephs) are localized on the axonal growth cones and perisynaptic astrocyte processes. This occurs during development and throughout adulthood (Ethell et al., 2001; Cahoy et al., 2008; Carmona et al., 2009; Filosa et al., 2009). Eph-Ephrin signaling is primarily known for its involvement in contact-mediated repulsion of the growth cone but also influences other physiological functions such as cell proliferation, migration, neurite extension and branching, regeneration, vascular development and even apoptosis in the developing brain (Murai and Pasquale, 2011). In the spinal cord, a specific subset of ventral astrocytes expresses semaphorin-3a (sema3a) that is involved in repulsion of the growth cone (Bagnard et al., 1998; Molofsky et al., 2014). In conclusion, astrocytes secrete axonal guidance molecules and can express its receptors, but the mechanisms by which they affect axonal outgrowth during development deserve further investigation.

## The Influence of Glia on the Global Organization of Neuronal Networks

At times of ongoing neuronal growth and remodeling, microglia and astrocytes have been described to be involved in essential developmental processes (Clarke and Barres, 2013; Ueno and Yamashita, 2014). Both cell types play crucial roles in either instructing neuronal cell death or in promoting survival with microglia being more involved in mediating cell death and astrocytes in neuronal survival. However, exceptions exist as well that will be discussed here.

### Control of PCD by Microglia

During early brain development, excessive numbers of neurons are formed, many of which undergo programmed cell death (PCD) starting prenatally with a peak in the postnatal period (Oppenheim, 1991; Thomaidou et al., 1997; Blaschke et al., 1998; Yeo and Gautier, 2004; Figure 1). This developmental mechanism is conserved in many species and occurs in several different neural cell types, including astrocytes (Krueger et al., 1995; Yeo and Gautier, 2004; Giffard and Swanson, 2005). As mentioned, microglia associate with dying neurons in a variety of CNS regions, such as the sub-plate and cortical layer II/III (Ferrer et al., 1990), hippocampus (Dalmau et al., 1997a; Wakselman et al., 2008), choroid plexus (Squarzoni et al., 2014), cerebellum (Marín-Teva et al., 2004), retina (Ashwell et al., 1989; Cuadros et al., 1992, 1993; Frade and Barde, 1998), optic nerve (Moujahid et al., 1996), and the spinal cord (Rezaie et al., 1999; Sedel et al., 2004; Calderó et al., 2009; Rigato et al., 2011). Also, microglia respond to PCD signals by engulfing dying neurons with their processes (Peri and Nüsslein-Volhard, 2008).

Besides their phagocytic activity, microglia can be actively involved in triggering neuronal death via the secretion of soluble factors or contact-mediated signals (Marín-Teva et al., 2004). For example, microglia can direct cells to apoptosis via secretion of NGF (Frade and Barde, 1998), tumor necrosis factor alpha (TNFα; Sedel et al., 2004; Taylor et al., 2005; Bessis et al., 2007), and the production of reactive oxygen species (ROS) via CD11b integrin and DAP12 immunoreceptor signaling (Wakselman et al., 2008). In addition, microglia can colonize the cortical proliferative zones where they can phagocytose neural progenitor cells, thereby controlling the production of neurons and macroglia in the developing cerebral cortex (Cunningham et al., 2013).

Although astrocytes are generally documented as neuroprotective cells as described in “Positive Effects of Microglia and Astrocytes on Neuronal Survival” Section (Banker, 1980; Vaca and Wendt, 1992), they too were shown to be capable of promoting apoptosis and neuronal cell death (Shute et al., 2005; Manoharan-Valerio et al., 2013). For example, in early postnatal hippocampal cultures, astrocytes induced cell death by electrical inhibition of neurons (Shute et al., 2005). It remains unclear whether in this case astrocytes remove neuroprotective or rather secrete apoptotic signals. Shute et al. (2005), after performing heat inactivation experiments, suggest that astrocytes secrete a heat-labile factor of unknown identity that can induce apoptosis in immature neurons. Further studies should be performed in order to investigate how and when astrocytes switch to inducing cell death, as they generally promote neuronal survival.

### Positive Effects of Microglia and Astrocytes on Neuronal Survival

Newborn neurons require trophic support during their assembly into neural circuits (Oppenheim and Johnson, 2003). Neurons lacking support from their environment fail to integrate into the developing neural network and undergo PCD. Apart from a role for microglia in PCD, they seem to be necessary for the survival of layer V cortical neurons during postnatal development, likely via the secretion of insulin-like growth factor 1 (IGF1) and other survival factors (Ueno and Yamashita, 2014). Growth factor colony-stimulating factor 1 (CSF1) and CSF1R are also thought to positively affect the survival of newborn neurons (Pollard, 2009; Ueno et al., 2013). Generally, CSF1 regulates maintenance and survival of microglia (Sawada et al., 1990; Suzumura et al., 1990; Ginhoux et al., 2010) and CSF1R is highly expressed by microglia during brain development (Sasmono et al., 2003; Bulloch et al., 2008). Whereas CSF1R deficiency in the CNS leads to abnormal brain development (Michaelson et al., 1996), CSF1 deficiency does not cause significant phenotypic changes in the brain. This discrepancy can be explained by the fact that apart from CSF1, interleukin-34 (IL-34) is another ligand for the CSF1R (Lin et al., 2008) that can successfully activate the CSF1R and its downstream pathways. Thus, CSF1R signaling, via its two ligands, plays a crucial role in the regulation of neural progenitor proliferation, differentiation and survival (Nandi et al., 2012). *In vitro* studies show that CSF1 stimulates neuronal outgrowth and survival only in neuronal cultures containing microglia and not in pure neuronal cultures (Michaelson et al., 1996). The involvement of CSF1R expressing microglia in the regulation and survival of both microglia and neurons advocates for a crucial role of CSF1R/CSF1 signaling mediated by microglia in proper brain development.

Neurons cannot survive without a close association with astrocytes. During development and throughout adulthood, astrocytes play important role in brain energy metabolism, K+ buffering and neurotransmitter recycling (Nedergaard et al., 2003; Sofroniew and Vinters, 2010; Bélanger et al., 2011). One function of astrocytes needed for neuronal survival is the scavenging of extracellular reactive oxygen species (ROS; Drukarch et al., 1998). Also, astrocytes express the excitatory amino acid transporters (EAAT) 1 and 2 which are involved in the clearance of glutamate from the synaptic cleft (Bjørnsen et al., 2014). By maintaining low extracellular glutamate and ROS, astrocytes prevent neurotoxicity. Moreover, astrocytes have been repeatedly reported to positively affect neuronal survival and neurite outgrowth via the secretion of a variety of neurotrophic factors such as TNFα, epidermal growth factor (EGF), ciliary neurotrophic factor (CNTF), bone morphogenetic proteins (BMPs), BDNF and somatostatin, with often the highest levels of expression during the period of early brain development (Shinoda et al., 1989; Schwartz and Nishiyama, 1994; Schwartz et al., 1996; Chang et al., 2003; Park et al., 2006).

For many astrocytic secreted factors, it is not clear how they promote neuronal survival, be it via stimulating neuron development (e.g., synapse formation as described in “Astrocytes Direct Postnatal Synaptogenesis” Section), or via triggering of neuron survival pathways. One of these factors is the neural and astroglial expressed neuropeptide somatostatin, which was initially described as neurotransmitter and neuromodulator (Schwartz et al., 1996; Koronyo-Hamaoui et al., 2011). Together with its receptors, somatostatin is highly expressed throughout the brain, mostly prenatal and during the first weeks after birth, i.e., at the time of astrogenesis (Hösli et al., 1994; Schwartz et al., 1996). Overexpression of somatostatin in astrocytes during brain development caused altered motor activity in mice, possibly caused by changes in the number of neurons and circuit wiring (Schwartz et al., 1996).

Another interesting factor is Erythropoietin (EPO), a hematopoietic factor that induces neural stem cell differentiation into astrocytes (Lee et al., 2004) but also stimulates astrocytes to produce growth factors, thereby promoting neuronal cell differentiation (Park et al., 2006). Some secreted factors have dual roles, such as interleukin-6 (IL-6), a neuropoetic cytokine produced by neurons and astrocytes in the brain where it exerts diverse functions. Astrocytic Il-6 stimulates neuronal differentiation during CNS development and in cultured neural progenitor cells from adult hippocampus (Gadient and Otten, 1997; Oh et al., 2010), while it promotes cell death during pathological situations and chronic IL-6 production reduces adult hippocampal neurogenesis (Van Wagoner and Benveniste, 1999; Vallières et al., 2002; Erta et al., 2012).

Thus, both microglia and astrocytes are essential cells taking part in the establishment and maintenance of neuronal networks by instructing neuronal cell death or promoting survival. In the next chapter, their role in the formation of local synaptic circuits will be discussed.

## Glia Control the Formation of Local Synaptic Circuits

Apart from their global roles in neural patterning, microglia and astrocytes can influence the formation and destruction of local synaptic circuits. Microglia are generally considered the “bad guys”, that prune synapses, and astrocytes the “good guys”, that induce synapse formation and save synapses from being eliminated. Also here, exceptions exist and astrocytes can mediate activity-dependent pruning and microglia can stimulate synaptogenesis under certain circumstances (Kettenmann et al., 2013).

### Can Microglia Stimulate Synaptogenesis?

The first wave of synaptogenesis occurs in rodents around embryonic day 14 (E14), when microglia are the only glial cells present (Figure 1). It has been suggested that at these early stages, microglia facilitate and promote synaptogenesis through the secretion of growth factors (Kettenmann et al., 2013), in contrast with their later roles in synaptic pruning (Bialas and Stevens, 2012). Microglia were found to secrete matricellular thrombospondins (TSPs), BDNF and anti-inflammatory cytokines that can promote neural survival as well synapse formation (Chamak et al., 1994, 1995; Lim et al., 2013; Parkhurst et al., 2013). Roumier et al. (2008) showed that prenatal microglia deficiency in mice having a loss-of-function mutation in DAP12 (DAP12KI mice) leads to synaptic dysfunction in the adult. DAP12 is a microglial signaling molecule of which mutations underlie Nasu-Hakola disease, as will be discussed in chapter 8. It is likely that the synaptic dysfunction seen here is caused by the inflammatory phenotype of the DAP12 mutated microglia. Namely, DAP12KI microglia overexpress several genes coding for inflammatory proteins such as interleukin-1β (IL1β), IL-6 and nitric oxide synthase 2 (NOS2). Moreover, similar effects were obtained after prenatal LPS induced immune activation of microglia. Thus, from this study, it can only be concluded that prenatal and immunologically activated microglia can have a negative effect on adult synaptic connections. Recently, it has been shown that in the developing somatosensory cortex, microglial contact with dendrites can induce synapse formation, mediated by calcium influx and actin accumulation at the contact site (Miyamoto et al., 2016). It is still largely unclear whether microglial contact was specific and necessary for the observed synapse formation. Also, the survival rate and functionality of the newly formed synapses needs further investigation. Nevertheless, also this study sheds light on immune regulation of neuronal circuit development.

### Neurocircuit Refinement—Pruning by Microglia and Astrocytes

Similar to the deleterious effects of microglia during remodeling of the overall landscape of the nervous system, local neural circuit refinement takes place through selective elimination of synapses and axon branches—a process named pruning (Chechik et al., 1998). Both microglia and astrocytes have been shown to play roles in developmental synaptic and axonal pruning by phagocytizing inappropriate synaptic connections and axons, thereby shaping neural circuits in the developing brain (Peri and Nüsslein-Volhard, 2008; Bialas and Stevens, 2012; Schafer et al., 2012; Schafer and Stevens, 2013). The first study demonstrating phagocytic microglia and astrocytes in the developing brain describes axonal pruning in cats (Berbel and Innocenti, 1988). Using light and electron microscopy these pioneers visualized large-scale axonal pruning of the developing corpus callosum. During the developmental pruning window, callosal axon degeneration was increased and both microglia and astrocytes contained axonal material within their cytoplasm.

For both types of glial cells, developmental pruning is an activity-dependent mechanism but the precise molecular pathways underlying synapse elimination remain largely unclear. One mechanism that was shown to mediate microglial synapse elimination in the developing CNS is the activation of the classical complement pathway (Stevens et al., 2007; Chu et al., 2010; Schafer et al., 2012). The complement cascade initiating protein q (C1q) localizes to developing synapses and microglia phagocytose these “tagged” synapses in a complement component 3 (C3) dependent manner (Stevens et al., 2007). It was proposed that immature astrocytes regulate C1q expression at synapses in need of elimination by the secretion of TGF-β (Stevens et al., 2007; Bialas and Stevens, 2013). Also, C1q−/− mice displayed enhanced synaptic connectivity and epileptic features (Chu et al., 2010), likely reflecting insufficient synaptic pruning. The complement cascade shifts microglia towards a proinflammatory phenotype which is manifested by the release of IL-6, TNFα, NO, an oxidative burst and increased phagocytic activity (Ilschner et al., 1996; Webster et al., 2000; Färber et al., 2009).

Regarding astrocytes, it has been proposed that they use different phagocytic pathways than microglia. Chung et al. (2013) demonstrated the involvement of the phagocytic receptors Multiple EGF-like-domains 10 (MEGF10) and MER Tyrosine Kinase (MERTK), both highly expressed in developing astrocytes, in astrocytic activity-dependent pruning that is independent of the complement factor C1q. They demonstrated *in vitro*, using purified immunopanned astrocytes (see Foo et al., 2011), and *in vivo*, using an Aldh1L1-EGFP2 transgenic mouse line, that the engulfment ability of Mertk−/− astrocytes was reduced as compared to wild type astrocytes. Moreover, both MEGF10 and MERTK deficient mice did not show refinement of the circuit, which was visible by an excess of functional synapses. They proposed that phosphatidylserine, recognized by MEGF10 and MERTK receptors, might serve as a tag for synapse elimination comparable to C1q for microglia (Hochreiter-Hufford and Ravichandran, 2013). It must be noted that MERTK is also expressed by microglia. Interestingly, microglia in Mertk−/− mice displayed an increase in synapse engulfment, possibly reflecting a compensation mechanism for the reduction in synaptic pruning by astrocytes (Chung et al., 2013). Thus, it seems that MERTK is dispensable for the phagocytic capabilities of microglia, where it is necessary for astrocytes.

### Astrocytes Direct Postnatal Synaptogenesis

After a period of synaptic pruning, synaptogenesis starts again which coincides with the peak of astrogenesis. As described previously, protoplasmic astrocytes engulf synapses by which they influence homeostasis (e.g., ion and pH regulation), metabolism (e.g., ATP and glucose supply) and neuronal activity (e.g., neurotransmitter regulation; Araque et al., 1999; Clarke and Barres, 2013). Because astrogenesis and synaptogenesis happen simultaneously and protoplasmic astrocytes are active participants of synapses, it is not surprising that they influence the establishment and function of synapses during development (Kucukdereli et al., 2011).

Astrocytes secrete several factors that can either drive or inhibit synapse assembly, such as the synaptogenic proteins TSPs and hevin (also known as secreted protein acidic and rich in cysteine (SPARC-like 1)) and the antisynaptogenic protein SPARC (Allen, 2013; Clarke and Barres, 2013). The driving capacity of astrocytes on excitatory synapse formation has been extensively studied in primary cultures of retinal ganglion cells, spinal motor neurons, and hippocampal neurons (Meyer-Franke et al., 1995; Pfrieger and Barres, 1997; Ullian et al., 2001; Kaech and Banker, 2006). In absence of astrocytes, cultured retinal ganglion cells (RGCs) show no or little synapse activity, whereas in their presence, RGCs formed more synapses and displayed increased synaptic activity (Meyer-Franke et al., 1995; Pfrieger and Barres, 1997). Apart from astrocytic support on the formation of glutamatergic synapses, astrocytes have also been shown to promote the formation of inhibitory synapses (Elmariah et al., 2005).

Besides controlling synapse number, astrocytes are essential for the function and efficacy of neuronal synapses (Allen, 2013). Astrocytes regulate presynaptic strength by the secretion of cholesterol, which promotes presynaptic activity and the probability of neurotransmitter release leading to increased dendrite differentiation (Mauch et al., 2001) and the promotion of postsynaptic strength by secretion of glypicans that facilitate postsynaptic insertion of glutamate receptors (Allen et al., 2012). In some cases, physical contact between astrocytes and neurons is necessary for neurons to be receptive for the synaptogenic signals produced by astrocytes (Hama et al., 2004; Barker et al., 2008). It was shown that protein kinase C (PKC) signaling is crucial for the local contact between astrocytes in embryonic hippocampal neurons (Hama et al., 2004). PKC is further thought to activate cellular processes that affect neuronal maturation processes, such as intracellular regulation of adhesion proteins needed for synapse formation. Another group examined contact-mediated synapse formation in developing RGCs and found that astrocytes in contact with RGCs induce synapse formation independent of PKC signaling. Astrocyte-RGC contact caused a translocation of the synaptic adhesion molecule neurexin away from dendrites (Barker et al., 2008). Neurexin is an inhibitor of synapse formation by interacting with the postsynaptic adhesion molecule neuroligin (Taniguchi et al., 2007) and thus translocation of neurexin is thought to promote synapse formation.

Excitatory synapses are surrounded by astrocytes more often than inhibitory synapses. This led to the suggestion that neural activity or the intracellular glutamate concentration of a synapse stimulates astrocyte process extension towards synapses (Genoud et al., 2006; Lushnikova et al., 2009; Bernardinelli et al., 2014a). However, astrocytic coverage of a synapse leads to synapse stabilization, which allows the synapse to mature further subsequently leading to higher intracellular glutamate concentrations (Bernardinelli et al., 2014b). For now, it can only be concluded that bidirectional interaction between synapses and astrocytes seems to be crucial for synapse coverage and maturation. Additional studies are needed to answer the following open questions; does developmental coverage of synapses by astrocytes depend on the brain region, type or activity of the synapse? Which molecular mechanisms cause astrocytes to extend their processes to synapses and is this driven by astrocytic intrinsic mechanisms or do astrocytes respond to signals from neurons? Answering these questions will contribute to the understanding of synaptic transmission. Well-regulated synaptic transmission has been shown to be crucial for learning and memory processes and deficiencies have been linked to several psychiatric and neurological disorders (van Spronsen and Hoogenraad, 2010).

## Is There a Causal Role for Glia in Neurodevelopmental Disorders?

Microglia and astrocyte abnormalities have been found in many pathologies, such as neurodevelopmental, neurodegenerative, autoimmune, affective disorders and also infectious diseases (Hanisch and Kettenmann, 2007; Ransohoff and Perry, 2009; Delpech et al., 2015). However, it is often not clear if these abnormalities are a cause or a consequence of the pathology (Rajkowska and Miguel-Hidalgo, 2007; Sofroniew and Vinters, 2010; Frick et al., 2013; Green and Nolan, 2014; Verkhratsky et al., 2014). Discussion of the many pathologies that could be caused or mediated by glia dysfunction is beyond the scope of this review (see for more detailed articles the references above). In this review article, several developmental functions of both microglia and astrocytes have been discussed. Here, we propose a possible causal role, rather than a purely reactive one, for both cell types in some neurodevelopmental disorders. Table 1 summarizes some of the main developmental mechanisms that are affected by glia and can be linked to neurodevelopmental disorders.

**Table 1:** **Glia dysfunction that affect developmental processes linked to neurodevelopmental disorders**.

Method of modulation	Developmental processes affected	Behavioral changes	Link to neurodevelopmental disorders	Reference
**Transient reduction**	Syn. pruning	Repetitive behavior	ASD	Zhan et al. (2014)
**in microglia**	Syn. transmission	Social interaction	Schizophrenia	Paolicelli et al. (2011)
CX3CR1 KO	Functional connectivity
**Microglia depletion**	DA axonal outgrowth	*Not defined*	Schizophrenia	Squarzoni et al. (2014)
CSF-1R blockade	Interneuron		ASD
Pu.1^−/−^ embryo’s	positioning
**Microglia deficiency**	Dendrito- and	Rett-like neurological	Rett syndrome	Chen et al. (2001)
MeCP2 KO	synaptogenesis	symptoms		Ballas et al. (2009)
				Maezawa et al. (2009)
**Microglia deficiency**	Synaptogenesis	Cognitive impairment	Nasa-Hakola	Roumier et al. (2004)
DAP12 KO	Syn. transmission		disease/PLOSL	
**Astrocyte deficiency**	Dendritogenesis	Rett-like neurological	Rett syndrome	Maezawa et al. (2009)
MeCP2 KO		symptoms
**Astrocyte deficiency**	Dendritogenesis	Fragile-X like	Fragile-X syndrome	Jacobs et al. (2010)
Fmr1 KO	Syn. function	behavior: seizures,		
	Neur. excitability	hyperactivity, learning		Higashimori et al. (2013)
		impairment
**Astrocyte modulation**	LTP	Enhanced fear	Depression	Nishiyama et al. (2002)
S100β KO	Syn. plasticity	memory	Schizophrenia
**Astrocyte modulation**	D-serine regulation	Anhedonia	Schizophrenia	Ma et al. (2013)
DISC1 mutant	NMDA	Cognitive impairments
	neurotransmission

*Abbreviations: syn., synaptic; neur., neuronal; DA, dopaminergic; KO, knockout; OE, overexpression; LTP, long term potentiation; ASD, autism spectrum disorder; OCD, obsessive compulsive disorder; PLOSL, polycystic lipomembranous osteodysplasia with sclerosin leukoencephalopathy*.

Mice harboring mutations in genes involved in glial-specific pathways are used in order to resolve questions about the causal role and consequences of glial cell dysfunction on neuronal function. For example, as described in “The Development and Distribution of Embryonic Microglia” Section, mice lacking CX3CR1, a receptor expressed only by microglia, have a transient reduction of microglia numbers specifically during the early postnatal period, a time during which microglia-mediated synaptic pruning is high (see “Positive Effects of Microglia and Astrocytes on Neuronal Survival” Section; Paolicelli et al., 2011). CX3CR1 deficient mice display deficits in synaptic pruning associated with weak synaptic transmission and reduced functional connectivity between several brain areas (Zhan et al., 2014). Moreover, these mice displayed deficits in social interaction and increased repetitive-behavior, an autism spectrum disorders (ASD)-like phenotype. These data suggest that disruptions in microglia-mediated synaptic pruning during a limited period of postnatal development could be a primary cause of some neurodevelopmental disorders.

An important feature of neurodevelopmental disorders, such as ASD and obsessive compulsive disorder (OCD), is an abnormal functional connectivity between brain areas (Harrison et al., 1998; Courchesne and Pierce, 2005; Schipul et al., 2011; Dichter, 2012; Jung et al., 2013). Abnormal functional connectivity can be caused by changes in synapse formation, function, and or abnormal synaptic pruning, which are also associated with developmental disorders (Derecki et al., 2012; State and Šestan, 2012; Tang et al., 2014; Washbourne, 2015). Also, variations in genes encoding for synaptic scaffolding proteins and proteins associated with synaptic transmission across cortical regions have been linked to neurodevelopmental disorders (Bourgeron, 2009; Kumar et al., 2011; Voineagu et al., 2011). As we have described in this review, both microglia and astrocytes play important roles in synaptogenesis and pruning and are thus, not surprisingly, found to be involved in the pathogenesis of neurodevelopmental disorders.

Another example of ASD is Rett syndrome, caused by mutations in the X-linked methyl-CpG binding protein 2 (MeCP2) gene. Patients, usually female, display dendritic and synaptic abnormalities (Zoghbi, 2003; Chahrour and Zoghbi, 2007). Mouse models with a loss-of-function mutation in *MeCP2* display neurological symptoms similar to human (Chen et al., 2001; Guy et al., 2001). Apart from the cell autonomous effect of non-functional MeCP2 proteins in neurons (Chen et al., 2001), there seems to be a non-cell autonomous effect of MeCP2-deficient glia on the changes in neuronal morphology (Ballas et al., 2009). Both astrocytes and microglia contribute to the pathology in genetic mouse models of Rett syndrome. *In vitro* studies with MeCP2-null microglia demonstrate that these microglia secrete neurotoxic high levels of glutamate thereby inducing dendritic and synaptic abnormalities (Ballas et al., 2009; Maezawa and Jin, 2010). MeCP2-null astrocytes further caused deficiencies in BNDF regulation, cytokine production, and abnormalities in dendritogenesis (Maezawa et al., 2009) whereas re-expression of MeCP2 in astrocytes could partially rescue the phenotype in global MeCP2 deficient mice. Also, the introduction of wild type BM into MeCP2−/− mice, which, according to the authors, lead to engraftment of wild type microglia, rescued the phenotype (Lioy et al., 2011; Derecki et al., 2012). However, recently, when Wang et al. (2015) aimed to replicate these results they did not find any amelioration of neurological deficits or prevention of early death in three different Rett syndrome models after introduction of wild type BM. Further research is thus necessary to investigate the role of glia in the etiology of Rett syndrome.

Mouse models of another type of ASD, i.e., Fragile-X syndrome, demonstrated the involvement of deficient astrocytes in its etiology (Jacobs et al., 2010). Fragile-X syndrome is caused by a mutation in the fragile X mental retardation 1 (*FMR1*) gene (Bakker et al., 1994). By co-culturing healthy neurons on fragile-X mutant astrocytes (*Fmr1−/−)*, it was shown that the presence of mutant astrocytes caused a delay in neuronal dendrite maturation and abnormal synaptic protein expression in healthy neurons (Jacobs et al., 2010). Whereas *in vivo* studies on the effects of fragile-x mutant astrocytes on developmental processes are still lacking, astrocytes were shown to express *Fmr1* during brain development (Pacey and Doering, 2007) and global *Fmr1−/−* mice display alterations in dendritic spine morphology, synaptic function and behavioral disturbances, similar to humans (Comery et al., 1997; Irwin et al., 2000; Nimchinsky et al., 2001).

Another disease proposed as a gliopathy, is Polycystic lipomembranous osteodysplasia with sclerosin leukoencephalopathy (PLOSL) also known as Nasa-Hakola disease (Hakola, 1972). Patients display bone abnormalities, axonal demyelination, astrogliosis, presenile dementia, and psychotic symptoms, similar to schizophrenia (Verloes et al., 1997; Paloneva BM et al., 2001; Satoh et al., 2011). The disease is caused by mutations in the transmembrane polypeptide DAP12 and one of its receptors triggering receptor expressed on myeloid cells 2 (TREM2). DAP12 and TREM2 are in the CNS only expressed in microglia and some oligodendrocytes (Schmid et al., 2002). DAP12 deficient mice display synaptic alterations, enhanced hippocampal LTP and changes in glutamatergic transmission lasting throughout adulthood (Tomasello et al., 2000; Roumier et al., 2004, 2008). These data demonstrate that prenatal modulation of microglial function can have long-term consequences on synaptic function and the development of Nasa-Hakola disease.

Dysfunction of dopaminergic circuits and displacement of inhibitory interneurons are other features of some neurodevelopmental and psychiatric disorders (Gonzalez-Burgos and Lewis, 2008; Tomassy et al., 2014). The previously described study from Squarzoni et al. (2014) points to a possible role of microglia in these developmental processes. Their data suggest a role for microglia in the control of dopaminergic axonal outgrowth and also the positioning of a subset of neocortical interneurons. Additional studies need to be done to investigate how exactly microglia contribute to axonal outgrowth and interneuron positioning, which molecular mechanisms are involved, and whether microglia deficiencies causing abnormalities in axonal tracts indeed lead to pathological behavior.

Much has been written about the role of mature reactive astrocytes in epileptogenesis, through their involvement in the maintenance of ion and neuron homeostasis (Wetherington et al., 2008; Coulter and Steinhäuser, 2015), but it is unclear whether astrocyte deficiencies during brain development could increase the susceptibility to develop epilepsy. It has been shown *in vitro*, using hippocampal slices, and *in vivo*, using a mouse model of astrogliosis without BBB breaching or other significant inflammation, that induction of astrogliosis can lead to hyper-excitability and epileptic seizures (Ortinski et al., 2010; Robel et al., 2015). Interestingly, neurodevelopmental disorders, in which astrocyte malfunction has been proposed as a causal factor, such as the above described Rett and fragile X syndrome, often go hand in hand with epileptic seizures (Jian et al., 2006; Hagerman and Stafstrom, 2009). Besides epilepsy, more traditionally seen “adult onset” psychiatric diseases, such as depression and schizophrenia, and other brain disorders, are now thought to have at least to some extent, a developmental and/or neuroinflammatory origin (Ansorge et al., 2007; Owen et al., 2011; Bozzi et al., 2012).

Various studies have implicated a role for astrocytes in the pathophysiology of schizophrenia, as reviewed in detail by Xia et al. (2016) and Mitterauer (2005). Two studies further imply a causal role for the dysfunction of astrocytes in the development of schizophrenia. First, an inducible astrocyte-specific Disrupted in schizophrenia 1 (DISC1) mutant mouse model was created (Ma et al., 2013). DISC1 is a protein that participates in the regulation of numerous cell functions such as cell proliferation, differentiation, migration and neurite outgrowth and has been linked to neurodevelopmental disorders (Brandon and Sawa, 2011). Astrocytes expressing mutant DISC1 were unable to regulate the production of D-Serine in the extracellular space which led to altered glutamatergic neurotransmission and changes in behavior such as anhedonia and cognitive impairments (Ma et al., 2013). Second, Nishiyama et al. (2002) investigated the effect of modulation of a major astrocytic protein S100β. *S100β* knockout mice displayed enhanced synaptic plasticity and performed better in contextual fear conditioning paradigms. These data were linked the structural changes seen in schizophrenia (Xia et al., 2016).

Overall we can conclude that, because both microglia and astrocytes are crucial in several developmental brain processes, it is reasonable to assume that glial deficiencies can lead to developmental abnormalities. Thus the glia could be part of the cause, and not only the consequence of neurodevelopmental disorders.

## Summarizing Statements

We reviewed the developmental roles of microglia and astrocytes (Figure 3). These cells are essential for brain function in health and disease, and their properties are conserved across species. Some of the remaining questions are summarized in Box 1. More insight in how glia develop and their functions during brain development will help understand their true contributions to several neurodevelopmental disorders.

Box 1 **Outstanding questions.**

Microglia invasion into the brain

- Which scaffold is used by the first microglia cells to enter the brain parenchyma before the establishment of the brain vasculature?
-When microglia enter the brain after the establishment of the brain vasculature, do they travel through the blood vessels, or do they use them as scaffold to migrate along into the brain parenchyma?
-Which signals are mediating the direction, speed and eventual distribution of microglia over the embryonic brain?

Astrocyte development and heterogeneity

- By which molecular signatures are distinct functional subtypes of astrocytes specified?
- Are astrocyte subtypes derived from distinct groups of progenitor cells and how do they develop to their diverse and complex morphologies?

Glia and developmental angiogenesis

- Which angiogenic molecules are expressed by microglia, when are theyexpressed, and how do they mediate developmental angiogenesis?
- Which aspects of the blood-brain-barrier are dependent on astrocytes and which ones become functional before astrocytes appear in the brain?

Glia and axonal outgrowth and guidance mechanisms

- Does the aligned structure of microglia along axonal tracts contribute to their functional association with axons?
- Are microglia able to directly affect axonal outgrowth?
- How do astrocyte-secreted axonal guidance molecules influence axonal outgrowth during development?

Astrocytes and programmed cell death (PCD)

- How and when do astrocytes switch from supportive functions that generally promote neuronal survival, to the induction of cell death?
- When promoting PCD, do astrocytes then function by removing neurotrophic factors, or rather by secreting apoptosis inducing factors?

Glia and synaptogenesis

- Which molecular mechanisms underlie the positive effects of microglia on the first wave of synaptogenesis?
- Does developmental coverage of synapses by astrocytes depend on the brain region, type or activity of the synapse?
- Which molecular mechanisms cause astrocytes to extend their processes to synapses and is this driven by astrocytic intrinsic mechanisms or do astrocytes respond to signals from neurons?

When comparing the developmental functions of microglia and astrocytes, two important distinctions can be made; their origin and timing of appearance in the CNS. Microglia are believed to derive from yolk-sac primitive macrophages and colonize the mouse brain during embryonic development as early as E8.5. Astrocytes derive from the neural lineage concurrently with the final stages of neurogenesis (Figure 1). Prenatally entering microglia most likely do so via extravascular routes, while at later stages, bone-marrow derived circulating macrophages might enter via the circulation. It remains unclear whether this happens only under inflammatory or also under physiological conditions. BM-derived macrophages that do enter the brain parenchyma do not seem to persist but rather fulfill their purpose and die or exit the brain.

Our understanding of the origin and development of astrocytes is still limited. The precise steps that neuroepithelium-derived progenitor cells undertake to become astrocytes are also unclear. Moreover, astrocytes are a heterogeneous population, and distinct subtypes are thought to have different developmental origins and functions. Astrocytic progenitors and subtypes are difficult to trace due to the lack of reliable, specific molecular markers. However, more molecular markers are emerging and this will contribute to a better understanding of astrocytes in the developing brain.

Embryonic microglia and newborn astrocytes are dynamic and motile cells that are located throughout the brain, but are also concentrated in specific hotspots. They can be found in close proximity of radial glial cells, dying cells, blood vessels, axons and synapses where they are thought to play part in neuro- and gliogenesis, neuronal cell death, angiogenesis, axonal outgrowth/pruning and synaptogenesis, respectively.

Both microglia and astrocytes influence neuro- and gliogenesis by affecting radial glia cell differentiation and functionality (Wiencken-Barger et al., 2007; Béchade et al., 2011; Cunningham et al., 2013). Also, both cell types are involved in neuronal cell death, although microglia are the key players in this developmental mechanism. Where microglia are the most important glial cells controlling developmental neuronal cell death and pruning, astrocytes positively affect neuronal cell survival and synaptogenesis. Nevertheless, exceptions exist as well that have been discussed in this review. For example, microglia are involved in the promotion of neuronal cell survival via the CSF1-CSFR signaling pathway and might positively affect prenatal synaptogenesis. Astrocytes are capable of inducing cell death and phagocytizing synapses tagged for elimination. The dual roles of both glial cells on synaptogenesis and synaptic pruning are likely dependent on the developmental stage of a certain brain area and the activation state of the glia.

Embryonic angiogenesis seems to be influenced by microglia which are in close association with developing vessels and are thought to secrete soluble factors stimulating vessel sprouting (Checchin et al., 2006; Rymo et al., 2011). So far it remains to be investigated how microglia are involved in embryonic angiogenesis and which factors are mediating this process. Astrocytes appear later in the brain and were shown to be necessary for postnatal angiogenesis in the retina and cortex, likely mediated by the secretion of VEGF (Stone et al., 1995; Ma et al., 2012). Moreover, astrocytes are involved in the formation of the BBB (Abbott, 2002).

In the case of axonal guidance, research is lacking in describing the precise roles of microglia and astrocytes in axonal outgrowth and guidance mechanisms. Microglia were shown to play part in the development of dopaminergic axons and the corpus callosum but understanding of the underlying mechanisms is lacking. Also, microglia might affect axonal outgrowth in a subtle, indirect way via the secretion of growth factors that affect PI3-kinase activity which was shown to be involved in axonal development, outgrowth and guidance. Astrocytes could affect axonal outgrowth due to their expression of extracellular matrix proteins laminin and fibronectin that are involved in contact-mediated attraction of the growth cone and axonal guidance molecules such as netrin-4, sema3a and ephrins. However, experiments are so far missing in which developmental expression of these genes is modulated in astrocytes, after which effects on axonal outgrowth could be investigated. Hence, the influence of both microglia and astrocytes on axonal guidance mechanisms deserves further investigation.

Finally, we have described examples of neurodevelopmental disorders in which the dysfunction of microglia and or astrocytes, that lead to abnormalities in developmental processes, is likely part of the cause and not merely a consequence of a certain underlying pathology.

In conclusion, glia are no longer considered “support cells” of the CNS whose function is to keep neurons “happy and together”. In fact, glia actively direct brain development in numerous ways. Neurons and glia work together in order to obtain proper neural development and brain function. Radial glia direct microglial migration, after which microglia control radial glia differentiation into neurons or astrocytes. Astrocytes, in turn, affect microglia maturation, radial glia and neuronal functionality. Thus, as a highly interactive network, the developing nervous system is strongly dependent on appropriate and coordinated signaling between neurons and its main types of glia.

## Author Contributions

KR is the first author of the review. SCN, PJL and EMH revised the work critically for intellectual content and contributed equally to the work. All four authors agree to be accountable for the content of the work.

## Conflict of Interest Statement

The authors declare that the research was conducted in the absence of any commercial or financial relationships that could be construed as a potential conflict of interest.
